# MicroEnv: A microsimulation model for quantifying the impacts of environmental policies on population health and health inequalities

**DOI:** 10.1016/j.scitotenv.2019.134105

**Published:** 2019-12-20

**Authors:** Phil Symonds, Emma Hutchinson, Andrew Ibbetson, Jonathon Taylor, James Milner, Zaid Chalabi, Michael Davies, Paul Wilkinson

**Affiliations:** a Institute of Environmental Design and Engineering, UCL, London, UK; b London School of Hygiene and Tropical Medicine, London, UK

**Keywords:** Microsimulation, Health modelling, Environmental risks, Deprivation, Air pollution, SDGs

## Abstract

The Sustainable Development Goals (SDGs) recognise the critical need to improve population health and environmental sustainability. This paper describes the development of a microsimulation model, MicroEnv, aimed at quantifying the impact of environmental exposures on health as an aid to selecting policies likely to have greatest benefit. Its methods allow the integration of morbidity and mortality outcomes and the generation of results at high spatial resolution. We illustrate its application to the assessment of the impact of air pollution on health in London. Simulations are performed at Lower Layer Super Output Area (LSOA), the smallest geographic unit (population of around 1500 inhabitants) for which detailed socio-demographic data are routinely available in the UK. The health of each individual in these LSOAs is simulated year-by-year using a health-state-transition model, where transition probabilities from one state to another are based on published statistics modified by relative risks that reflect the effect of environmental exposures. This is done through linkage of the simulated population in each LSOA with 1 × 1 km annual average PM_2.5_ concentrations and area-based deprivation indices. Air pollution is a leading cause of mortality and morbidity globally, and improving air quality is critical to the SDGs for Health (Goal 3) and Cities (Goal 11). The evidence of MicroEnv is aimed at providing better understanding of the benefits for population health and health inequalities of policy actions that affect exposure such as air quality, and thus to help shape policy decisions. Future work will extend the model to integrate other environmental determinants of health.

## Highlights

• We describe a microsimulation model for quantifying environmental risks to health.
• The model outputs temporal health impacts at a high spatial resolution.
• Our approach allows the integration of estimated morbidity and mortality impacts.
• The model may be used to assess health impacts of air pollution policy.
• Multiple environmental exposures may be overlaid with SDGs in mind.

## Graphical abstract

**Figure:**
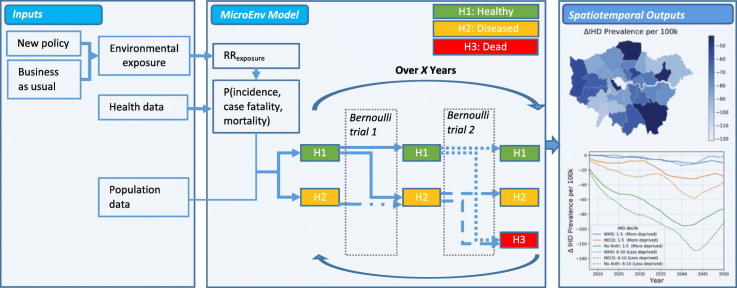

## 1 Introduction

Reducing the adverse consequences of poor environmental conditions is an important objective of several of the Sustainable Development Goals (United Nations, 2017), including, but not limited to, Goal 7 (affordable, clean energy), Goal 11 (sustainable cities and communities), and Goal 13 (climate action). Policies aimed at improving environmental conditions also have benefits to health, either through direct effects or as co-benefits (such as increasing physical activity). Therefore, excess mortality due to air pollution is used as one of the indicators of health under SDG 3 (health, well-being). Quantifying the potential impact on health of policies aimed at achieving these SDGs, or of reducing environmental exposures in general, is of increasing interest to policy-makers in order to track progress and evaluate effectiveness of policies and their respective impacts on health. Such policies include those aimed at the transition to a low carbon economy, many of which have potential impacts that are often, but not always, beneficial for health. Among the prominent issues of concern are those of outdoor air pollution and population health.

Substantial epidemiological evidence has shown the large impact that air pollution has on population health (Brunekreef and Holgate, 2002). The World Health Organisation (WHO) has estimated an annual impact of air pollution to be around 7 million premature deaths worldwide, with 3 million of these attributable to outdoor air pollution (WHO, 2014), whilst the Lancet Commission on Pollution and Health puts the combined figure at 9 million (Landrigan et al., 2018). There is good evidence that people exposed to higher air pollution are at a higher risk of non-communicable diseases such as ischemic heart disease (IHD) and stroke. In the UK, it has been estimated that air pollution contributes to around 40,000 premature deaths (Royal College of Physicians, 2016). As exposure to air pollutants is generally higher in urban settings, the trend of rapid urbanization over recent decades has tended to increase burdens globally (United Nations, 2018). Air pollution may also often contribute to socio-economic inequalities in health (O'Neill et al., 2003). Inequalities within society in developed countries, such as the UK, can be seen to exacerbate poor health (Marmot, 2017). The importance of reducing air pollution exposures is reflected in the SDG indicators 7.1.2 (access to clean energy in homes), 11.6.2 (air quality in cities), and 3.9.1 (air pollution-related mortality).

Empirical scientific evidence is necessary to evaluate potential policy interventions which may help achieve these SDGs. Many methods of impact quantification are based on life-table methods (such as (Miller and Hurley, 2003)), or a combination of life-table and separate direct estimation of morbidity impact, as has been used by Hamilton et al. (2015) to assess the health impacts of energy efficiency retrofits to UK homes. These methods do not fully integrate morbidity and mortality modelling and mostly have not been applied with detailed segmentation of the population by area or demographic group.

Microsimulation methods have become an increasingly popular modelling tool for the use of health impact assessment (Rutter et al., 2011; Schofield et al., 2018). They allow public health policies to be evaluated through scenario modelling (Zucchelli et al., 2012) and can be used at high spatial resolutions where some data may be missing (Ballas et al., 2006; Smith et al., 2011). Building microsimulation models with multiple disease states allows quantification of illness as well as mortality, potentially including multiple co-morbidities. This was demonstrated by (Lymer et al., 2016) who looked at the co-morbidities of diabetes and cardiovascular disease (CVD) due to obesity in Australian adults. Previous studies have also assessed the burden of obesity on non-communicable diseases (NCDs) such as CVD and cancer (Webber et al., 2014) and in another application, the effect of deprivation on screening methods for CVD (Kypridemos et al., 2016).

In this work, we sought to develop a flexible modelling method (MicroEnv) capable of examining the impact of various environmental influences on the health of populations. The aim of the model is to be able to integrate evidence on both morbidity and mortality from multiple forms of exposures, at high spatial resolution, in order to assess the health impacts of changes in different environmental conditions. The objective of this paper is to describe the development of this model, based on microsimulation, and to demonstrate its application to investigate changes in population exposure to background PM_2.5_, and associated morbidity and mortality (including health inequalities) from Ischemic Heart Disease (IHD) in London, UK. We then consider the strengths and weaknesses of this modelling approach for assessing policy interventions, comparing this model against other models, and describing how such methods may be used to investigate the effectiveness of policies aimed at achieving the SDGs.

## 2 Methods

**Fig. 1:**
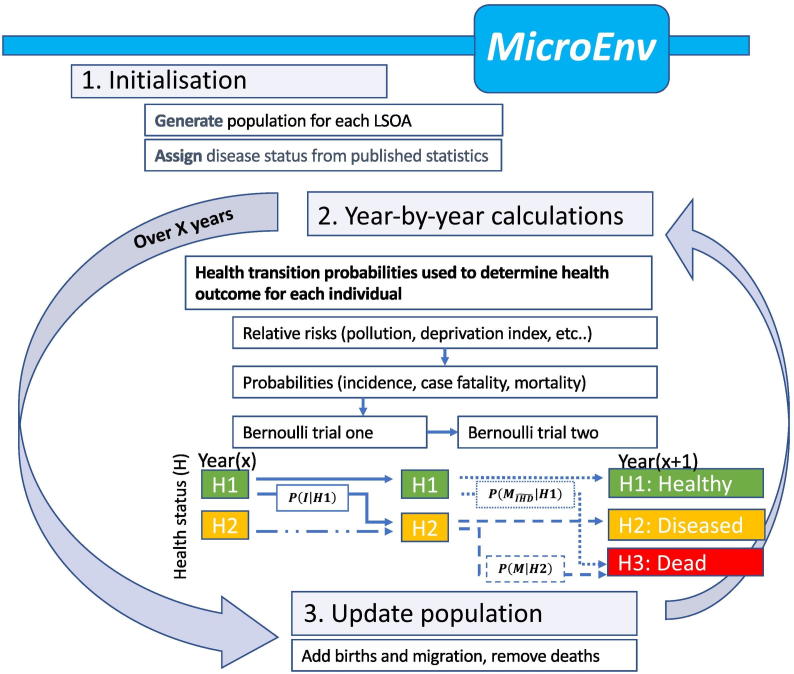
Schema of the microsimulation model.

Our microsimulation model simulates individuals at local area level and currently includes exposure to particulate air pollution and socio-demographic status coupled with population aging. In this paper we demonstrate its application for London, although the model has also been structured and parameterized for assessing health impacts in the population of Rennes, France. The model's general structure is shown in Fig. 1, and the data sources used in Table 1. Supplementary data (Appendices B and C) provide further details on the input data sources used in the model.

**Table 1:** Data sources and relative risks used in the microsimulation model.

Datasets used in MicroEnv			
Data type	Year	Additional info	Reference
Population	2015	Population by single year of age, gender and LSOA^a^	ONS (2018a)
Socio-economic deprivation	2015	Decile of the Index of Multiple Deprivation (IMD) for each LSOA	DCLG (2015)
Air pollution	2014	Annual averages of PM_2.5_ at 1 × 1 km grid (mapped to LSOA)	Ricardo Energy and Environment (2017)
General fertility rates	2015	Number of live births per 1000 females aged 15–44 at local authority level. Applied to the LSOA-specific female population each year	ONS (2018b)
Mortality (all-cause)	2016	Period projections by year of age and gender (UK)	ONS (2017)
IHD mortality, incidence and prevalence	2016	By gender and 5-year age bands (UK)	GBD Results Tool (Institute for Health Metrics and Evaluation, 2019)


Relative risks used in MicroEnv		
Relative risk	Coefficient used	Reference
IHD incidence	1.08 per 10 μg/m^3^ (PM_2.5_)	Wilkinson et al. (2018)
IHD case fatality	1.21 per 10 μg/m^3^ (PM_2.5_)	Wilkinson et al. (2018)
All-cause mortality	1.06 per 10 μg/m^3^ (PM_2.5_) 1.7 between most and least deprived decile (males) 1.5 between most and least deprived decile (females)	Pope III et al. (2002) ONS (2015) ONS (2015)

a LSOA – Lower Layer Super Output Area.

The first step is to generate the population for the region under investigation. Here, Greater London's initial population was defined for each Lower Layer Super Output (LSOA) level using an updated (2015) version of 2011 census data (ONS, 2018a). An LSOA is a geographic unit with, on average, a population of around 1500 inhabitants of which there are 4835 in Greater London. Each individual within an LSOA is generated, replicating its exact age and gender structure. Hence, around 8.5 million individuals are generated for the whole of Greater London. To incorporate morbidity into the model in the form of ischemic heart disease (IHD) prevalence, we specified a three-state transition model in which individuals may be in one of the following states:

H1 : being free of diagnosed ischaemic heart disease (IHD)

H2 : having been diagnosed with IHD (prevalent IHD)

H3 : dead from any cause.

At year zero of the simulation, a subset of the population is initialized as having diagnosed ischaemic heart disease using prevalence data at 5-year age bands for the UK in 2016 as the baseline (the latest available data, from the Global Burden of Disease (GBD) Results Tool (Institute for Health Metrics and Evaluation, 2019). This was also the source for the age and gender specific IHD incidence and mortality rates used in our model. The methods employed in constructing the GBD Results Tool is documented elsewhere (GBD 2017 Disease and Injury Incidence and Prevalence Collaborators, 2018). Briefly, GBD employ DISMOD-MR 2.1, a Bayesian meta-regression tool, used in combination with routine clinical data to estimate incidence, prevalence and mortality rates for various countries. Further details of the health data used in the model are provided in the supplementary data (Appendix B) including a comparison of the GBD Results Tool outputs to English Health Survey data.

The health state of each member of the simulated population is updated year-by-year by performing sequential Bernoulli trials (random experiments with two possible outcomes). For individuals in a non-diseased state (H1), an initial Bernoulli trial determines whether he/she develops disease. Following this, a second Bernoulli trial is performed to determine whether the individual dies (moves to H3 state). For individuals already in a diseased state, only the second trial is required. Note that we assume that once diagnosed with IHD, a person remains in the ever diagnosed IHD state(H2) until death, so that the probability of complete recovery to a state of being without diagnosed IHD is zero even though some people with IHD may become entirely asymptomatic following treatment. We are only required to calculate three health-state transition probabilities (as shown in Fig. 1). These are the:

• probability of incidence from a healthy state, *P*(*I*| *H*1),
• probability of mortality from a healthy state due to all but non IHD causes, $P\left(M_{\tilde{\mathrm{IHD}}}|H1\right)$,
• probability of mortality from a diseased state, *P*(*M*| *H*2).

### 2.1 Incidence probabilities

The health transition probabilities to new diagnosis of IHD, *P*(*I*| *H*1), are determined by age and gender specific incidence rates derived from the GBD Results Tool, which are reported per 100,000 of the *total* UK population. GBD outputs can then be converted to give the incidence probability for the total population (i.e. including in the denominator those with and without diagnosed IHD), *P*(*I*). Application of Bayes theorem (see supplementary data Appendix A for further details) can be used to calculate the health transition probability for incidence in those without diagnosed disease, *P*(*I*| *H*1), as:

(1) $$P\left(I|H1\right)=\frac{P\left(H1|I\right)P\left(I\right)}{P\left(H1\right)}$$

(2) $$P\left(I|H1\right)=\frac{P\left(I\right)}{\left(1-P\left(H2\right)\right)}$$

### 2.2 Mortality probabilities

Death rates (from any cause) in those with and without diagnosed IHD are derived from published statistics using two key assumptions: (i) that the death rate from non-IHD causes is equal in those with and without diagnosed IHD, and (ii) the simplifying assumption that IHD mortality can only occur in those with recognised (prevalent) IHD. In a later version of this model, we will alter this assumption to reflect the occurrence of sudden cardiac death in those without recognised IHD.

The transition probability to death (from all non-IHD causes) from a healthy state, $P\left(M_{\tilde{\mathrm{IHD}}}|H1\right)$, is again calculated by invoking conditional probabilities:

(3) $$P\left(M_{\tilde{\mathrm{IHD}}}|H1\right)=\frac{P\left(H1|M_{\tilde{\mathrm{IHD}}}\right)P\left(M_{\tilde{\mathrm{IHD}}}\right)}{P\left(H1\right)}$$

(4) $$P\left(M_{\tilde{\mathrm{IHD}}}|H1\right)=P\left(M_{\tilde{\mathrm{IHD}}}\right)$$

Here, $P\left(M_{\tilde{\mathrm{IHD}}}\right)$ is the probability of death from any cause other than IHD which is calculated by subtracting the age and gender specific IHD mortality probability from the all-cause mortality rates ($P\left(M_{\tilde{\mathrm{IHD}}}\right)=P\left(M_{\mathrm{All}-\mathrm{cause}}\right)-P\left(M_{\mathrm{IHD}}\right)$), as published by the Office for National Statistics (ONS) (ONS, 2017). This subtraction is performed following the application of the relative risks described 2.4, 2.5, thus, avoiding the double counting of mortality rate multipliers. Assuming that disease prevalence and other-cause mortality are considered independent events, $P\left(H1|M_{\tilde{\mathrm{IHD}}}\right)=P\left(H1\right)$ and $P\left(H2|M_{\tilde{\mathrm{IHD}}}\right)=P\left(H2\right).$ This means that baseline other-cause mortality rates are the same for those with and without disease.

The transition probability to death from diseased state is the sum of the conditional IHD mortality and other-cause mortality probabilities:

(5) $$P\left(M|H2\right)=P\left(M_{\mathrm{IHD}}|H2\right)+P\left(M_{\tilde{\mathrm{IHD}}}|H2\right)$$

(6) $$P\left(M|H2\right)=\frac{P\left(H2|M_{\mathrm{IHD}}\right)P\left(M_{\mathrm{IHD}}\right)}{P\left(H2\right)}+\frac{P\left(H2|M_{\tilde{\mathrm{IHD}}}\right)P\left(M_{\tilde{\mathrm{IHD}}}\right)}{P\left(H2\right)}$$

(7) $$P\left(M|H2\right)=\frac{P\left(M_{\mathrm{IHD}}\right)}{P\left(H2\right)}+P\left(M_{\tilde{\mathrm{IHD}}}\right)$$

The above is derived using the same reasoning as above, $P\left(H2|M_{\tilde{\mathrm{IHD}}}\right)=P\left(H2\right),$ which means: $\frac{P\left(H2|M_{\tilde{\mathrm{IHD}}}\right)}{P\left(H2\right)}=1$ and IHD mortality is assumed to only be possible from a prevalent state: *P*(*H*2| *M*_*IHD*_) = 1.

Finally, the probabilities for remaining in the same health state in a given year, are calculated via subtraction of the transition probability from unity. For example, the probability of non-incident disease is given by; $P\left(\tilde{I}|H1\right)=1-P\left(I|H1\right)$. The model allows projections of change in underlying mortality rates; however, a simplifying assumption is used which holds rates constant at those for the base year (2016). Once an individual has died, they are removed from any subsequent calculations.

### 2.3 Births and migration

Newborns are added to the simulated population each year using published 2015 UK ONS general fertility rates (GFR – live births per 1000 females aged 15–44 per year) provided at local authority level (ONS, 2018b). GFRs are applied to the size of the LSOA-specific female population aged 15–44 at the end of each simulation year. Further details are provided in supplementary data Appendix C. In the current implementation, migration of people from year-to-year (both within the city and into and out of it) is assumed to be zero. This simplifying assumption is in part made because of the complexity and uncertainties of having to update LSOA data each year based on a very large matrix of LSOA-to-LSOA migration probabilities. It is a reasonable assumption for short term assessments, but increasingly less secure for analyses over longer-term horizons.

### 2.4 Socio-economic deprivation

The effect of deprivation was taken into account by multiplying mortality rates by a relative risk derived from analyses published by the ONS (ONS, 2015) for England and Wales (map provided in supplementary data Appendix C). These data indicate a broadly linear relationship between decile of the Index of Multiple Deprivation (IMD) and mortality risk, where decile 1 is most deprived (representing the most deprived 10% of the population). In men, the risk of death in the most deprived decile of IMD was 1.7 times that of men in the least deprived decile; in women the corresponding figure was 1.5. Thus, to adjust the population average risk (at an average IMD decile of 5.5) for an LSOA of IMD decile, *j* (*j* = 1, 2,…10), we applied a relative risk as follows:

(8) $$\mathrm{RR}\left(\mathrm{depr},\mathrm{men}\right)=1.7^{\left(5.5-j\right)/9}$$

(9) $$\mathrm{RR}\left(\mathrm{depr},\mathrm{women}\right)=1.5^{\left(5.5-j\right)/9}$$

A relative risk for deprivation is also applied to IHD mortality rates, although not for IHD incidence due to lack of empirical evidence.

### 2.5 Air pollution

To quantify the impact of outdoor air pollution on IHD, we used evidence from review of the published epidemiological literature on the effect of air pollution on each of the key state transition probabilities (Wilkinson et al., 2018). We assumed that the effect of air pollution on the transition probability for diagnosis of IHD, *P*(*I*| *H*1), is represented by epidemiological studies of the relative risk of disease incidence in relation to the concentration of PM_2.5_ (which we refer to as *RR*(*incidence*)). The relative risk for death (from any cause) among those with a diagnosis of IHD, *P*(*M*| *H*2), was based on review of studies that reported the effect of air pollution on survival (‘case fatality’) in people following diagnosis of an IHD event (*RR*(*case fatality*)). For the current analyses we assumed these relative risks to be 1.08 and 1.21 per 10 μg/m^3^ increase in PM_2.5_, respectively.

The relative risk for the effect of air pollution on the overall risk for death from all causes was derived from published evidence, and assumed to be 1.06 for a 10 μg/m^3^ increase in PM_2.5_ (Pope III et al., 2002). This was used to derived a relative risk of mortality in those without recognised IHD adjusted to take account of the effect of air pollution on death among those with IHD.

PM_2.5_ concentrations used to compute the area-specific relative risks were based on 1 × 1 km grid modelled background air pollution concentrations for 2014 (DEFRA, 2015) using the ADMS pollution dispersion model. Further details are reported elsewhere (Ricardo Energy and Environment, 2017) and the annual average air pollution has been mapped at LSOA level and is presented in supplementary data Appendix C. Modelled gridded values were mapped to provide an average exposure for each LSOA. Annual average pollutant concentrations, *x*, at LSOA level are input into relative risk calculations, using the difference between *x* (base case and alternative scenarios) and the UK population weighted average concentration, *μ*. Relative risks are applied to all state transitions needed to estimate impact. For example, for disease incidence the relative risk of PM_2.5_ exposure would be calculated as:

(10) $$\mathrm{RR}\left(\mathrm{incidence}\right)=\mathrm{RR}{\left(\mathrm{PM}2.5,\mathrm{incidence}\right)}^{\left(x-\mu \right)/10}$$

Details of all relative risks used in the model are provided in Table 1.

#### 2.5.1 Scenarios modelled

To illustrate how the model may be used to derive estimates of the effect of potential interventions that alter the concentration of PM_2.5_, four simulation scenarios have been run, one with concentrations held at baseline (here 2014) levels, and three with altered levels reflecting theoretical interventions:

A) WHO: Compliance with World Health Organisation guidelines (WHO, 2005) (i.e. ambient outdoor PM_2.5_ annual mean does not exceed 10 μg/m^3^ – only affects LSOAs where PM_2.5_ > 10 μg/m^3^)
B) NECD (National Emissions Ceiling Directive): UK emission reductions in line with EU Directive 2016/2284/EU resulting in the baseline concentration reducing by 3.6 μg/m^3^ across Greater London (GLA, 2017)
C) No Anth: Complete removal of PM_2.5_ of anthropogenic origin.

An assumption is made that these interventions result in an instantaneous reduction in PM_2.5_ levels within effected LSOAS, taking place at simulation initialisation (year zero). Throughout the simulation, PM_2.5_ levels remain static with time. The impact of each scenario's change in air pollution exposure on mortality and IHD prevalence rates was then estimated using results from the three scenario model runs with respect to those in the base case scenario:

(11) $$∆\;\mathrm{IHD}\;\mathrm{Prevalence}=\frac{N_{\mathrm{diseased}}^{\mathrm{scenario}}}{N_{\mathrm{population}}^{\mathrm{scenario}}}-\frac{N_{\mathrm{diseased}}^{\mathrm{base case}}}{N_{\mathrm{population}}^{\mathrm{base case}}}$$

(12) $$∆\;\mathrm{Mortality}=\frac{N_{\mathrm{deaths}}^{\mathrm{scenario}}}{N_{\mathrm{population}}^{\mathrm{scenario}}}-\frac{N_{\mathrm{deaths}}^{\mathrm{base case}}}{N_{\mathrm{population}}^{\mathrm{base case}}}$$

In scenarios where PM_2.5_ emissions have reduced, a cessation lag is applied to the relative risks associated with mortality. This accounts for the fact that a reduction in PM_2.5_ doesn't lead to an immediate health benefit. For all-cause mortality, we have used the recommended lag from the US Environmental Protection Agency (US EPA, 2010). Whilst there is some uncertainty in the lag structure, a smooth function is used to reflect that 30% of the benefit of reduced PM_2.5_ occurs in the first year, 50% over years 2 to 5 and 20% over years 6 to 20. In the case of IHD mortality, an exponential decay curve is used, as informed by evidence on smoking cessation (Lightwood and Glantz, 1997).

### 2.6 Computation and model outputs

The simulation software is written in Python v3.6 (Van Rossum and Drake, 2011). Running annual Bernoulli trials for each of the 8 million population of London is a relatively computational-intensive process, generally requiring High Performance Computing (HPC) facilities to allow parallel processing of the populations of multiple (typically 100) LSOAs. The simulation for a typical LSOA produces a file of around 30 KB and takes around a minute to run for a 50-year simulation on a 2.9 GHz processor. This means that the simulations would take around three and a half days for Greater London if performed without parallel processing (longer for multiple policy scenarios). The model outputs a database, which contains the annual health status of each individual within the simulated population (both alive and dead). This data is stored in a compressed comma separated values (‘.csv.gz’) file for each LSOA. Parallel processing is again used to aggregate the data into age, gender, deprivation decile and year stratified output files – 240 KB for 100 LSOAs. A post-processing script is used to aggregate and plot the results; further analyses can then be undertaken as required to compute numbers of new cases of disease, the prevalent population, deaths, and years of life lost by population group and year. Outputs may also be processed in Geographical Information Systems (GIS) or statistical programming software.

## 3 Results

We illustrate the use of the model by its application by simulating the impact of air pollution on population heath in London, UK. Air pollution can be used as a marker of sustainable development, as measures taken to achieve a low-carbon economy, such as reductions in transport or industry impact on atmospheric emissions, and thus effect a reduction in air pollution, including climate modifying pollutants. Modelling the population impact of changes in air pollution on health, can thus be used to quantify the health co-benefits of the transition to a low carbon economy, and provide valuable insight for understanding where, and to whom, to target policy interventions in order to reduce air pollution-related morbidity and mortality. The base case is assumed to be exposure to 2014 concentrations of PM_2.5_; three theoretical counterfactual cases have been simulated as described in Section 2.5.1. In application to particular policy assessments, the counterfactual would usually be determined by assessing the corresponding change in concentrations that are likely to result for a particular policy using emission-dispersion modelling or similar, but here we simulate several PM_2.5_ reduction scenarios to illustrate the principle.

**Fig. 2:**
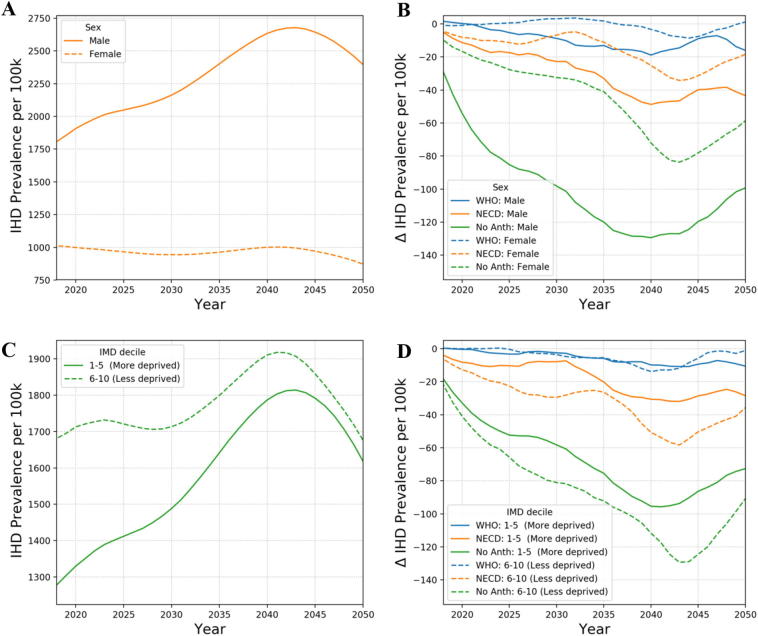
Simulation results for IHD prevalence per 100 k working age (15–64) population by calendar year (5-year running mean): A) by sex for the base case scenario (PM_2.5_ concentrations at 2014 level), B) the alternative scenarios – base case by sex, C) by deprivation for the base case scenario, D) the counterfactual scenarios – base case by deprivation.

**Fig. 3:**
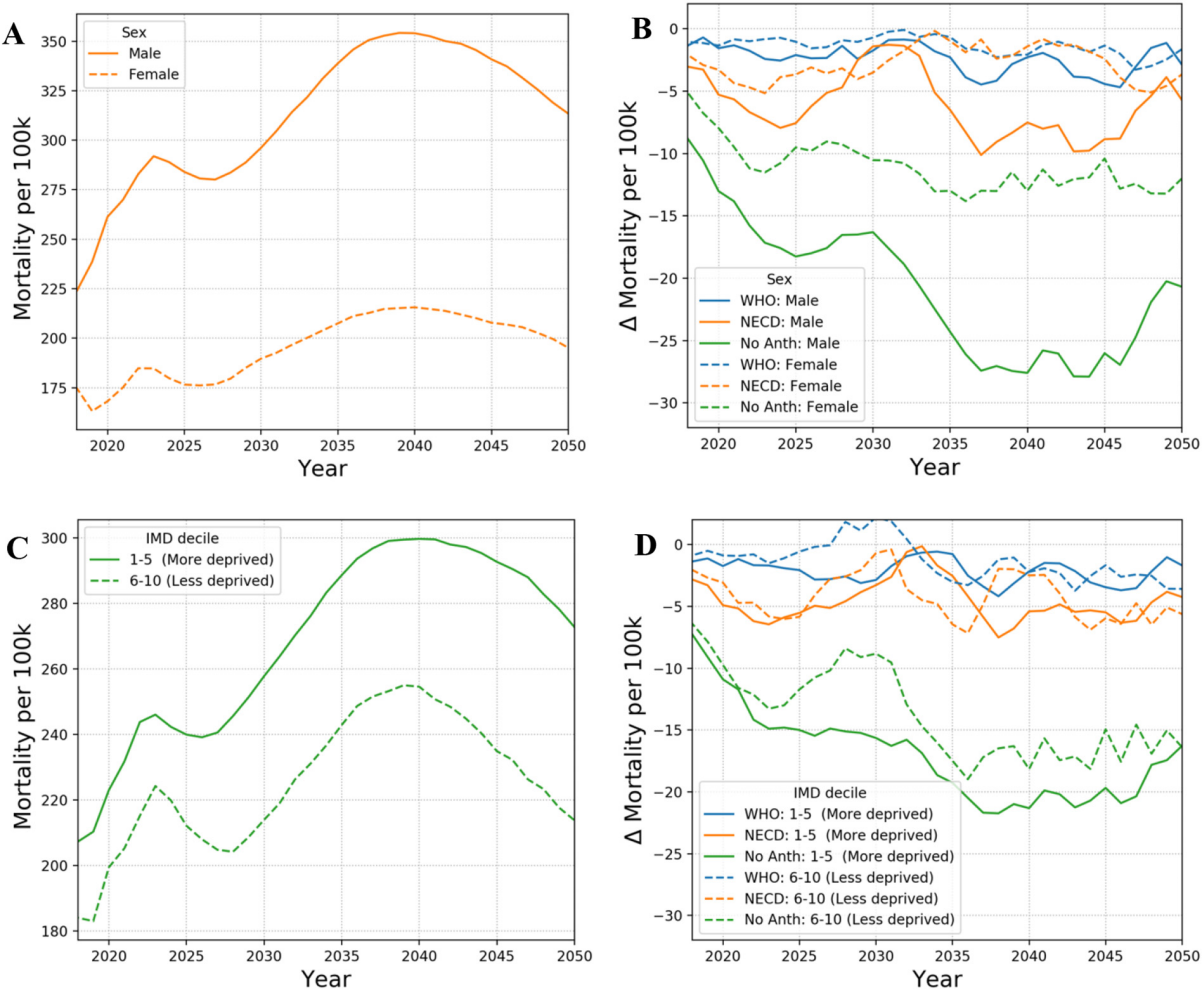
Simulation results for all-cause mortality rates per 100 k working age (15–64) population by calendar year (5-year running mean): A) by sex for the base case scenario (PM_2.5_ concentrations at 2014 level), B) the alternative scenarios – base case by sex, C) by deprivation for the base case scenario, D) the alternative scenarios – base case by deprivation.

Fig. 2 demonstrates the application of the model showing how IHD prevalence evolves over time under the base case scenario and also the impact of reducing particulate air pollution on the resulting change in IHD prevalence over time (as calculated using Eq. (11)). The results are shown for the working age population (15–64) and are grouped by gender and resident index of multiple deprivation (IMD). A 5-year running mean is used to remove statistical anomalies from particular years. The model results indicate that the removal of all anthropogenic PM_2.5_ air pollution has a beneficial impact on disease prevalence for individuals of working age. The NECD scenario where PM_2.5_ is reduced by 3.6 μg/m^3^ across the whole of Greater London leads to a greater health benefit than meeting the WHO guidelines of 10 μg/m^3^ (which only impacts on the more polluted areas of the city). Greater benefits are achieved for males then for females, which is due to the fact that males have higher underlying disease prevalence rates than women and so gain more from reduced pollution levels. The gradual improvement over time is in part due to the cessation lag programmed into the model but also because of the age demographics of London. London has a fairly young population with the population pyramid peaking at around 30 years of age. Prevalence increases as this age group ages with the maximum prevalence benefit due to reducing air pollution observed at around 2040. Maximal benefit is slightly earlier for males than females, since on average males tend to contract disease earlier. The results for deprivation follow a similar trend with the prevalence reduction peaking earlier for those living in more deprived areas than those in less deprived areas. This is again due to people in less deprived areas typically developing disease later in life. It is noted that prevalence in the base case run is higher in the less deprived group. This is because this demographic group is generally older at model initialisation. In Fig. 3, we show model results for the impact of reducing PM_2.5_ on all-cause mortality rates. Similar trends are observed as with the prevalence results, however, the more deprived population sub-group now gains most. A result of the deprivation relative risks applied within the model.

**Fig. 4:**
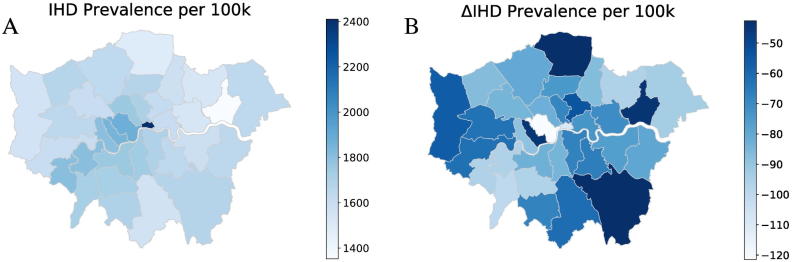
Illustration of Local Authority-level outputs for Greater London: IHD prevalence rates per 100 k working age (15–64) population. A) Under the base case scenario, and B) the change in IHD prevalence resulting from the removal of PM_2.5_ of anthropogenic origin. The results shown are averaged over the 2018–2050 modelling period.

**Fig. 5:**
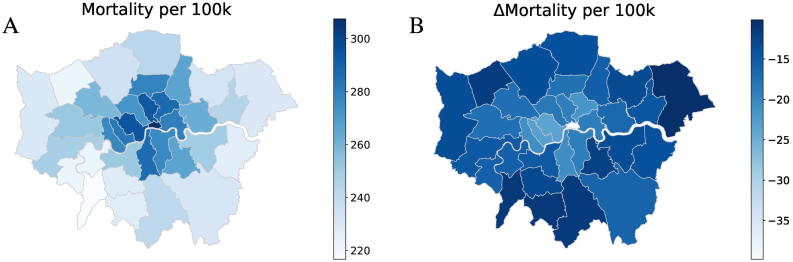
Illustration of Local Authority-level outputs for Greater London: all-cause mortality rates per 100 k working age (15–64) population. A) Under the base case scenario, and B) the change in all-cause mortality rates resulting from the removal of PM_2.5_ of anthropogenic origin. The results shown are averaged over the 2018–2050 modelling period.

Fig. 4, Fig. 5 show illustrative maps of the microsimulation predictions for IHD prevalence and all-cause mortality (per 100,000 population) under the base case scenario and also the change in rates after removing anthropogenic air pollution emissions (No Anth scenario). Maps for the other pollution reduction scenarios are provided in supplementary data Appendix D. Results are averaged over the period 2018–2050 for Greater London with LSOA results aggregated to Local Authority/borough level. Reductions in prevalence appear to be greatest in central (more polluted) and more affluent parts of London (south west). In these areas the average age of the population is toward the higher end of 15–65, which means base prevalence rates tend to be higher. Improvements in mortality rates are mainly focused toward more central boroughs where the reduction in pollution is greatest. Model results enable the impact of air pollution policy on social inequalities to be considered.

## 4 Discussion

This paper describes the mathematical and computational framework of the MicroEnv model and demonstrates its application to London to support the analysis of the population health impact of policies aimed at improving environmental exposures. The general framework may be adapted to cities across both developed and developing contexts, where underpinning data is available. Such models have particular value in helping to identify policies that provide the greatest potential benefit to health and health inequalities and might be used for quantification of the likely co-benefits from policy changes in areas such as transport, clean energy, waste management, and urban planning. We are currently developing the model to incorporate exposures in both the indoor and outdoor environment, as well as from physical activity to attempt to give a more integrated picture of the impacts on population health of combined actions relating to achievement of SDG goals.

Microsimulation is only one of a range of methods that might be used for such modelling and has a number of advantages over other methods. First, because it models the fate of *individuals* in the population, there is almost unlimited flexibility in how the results can be aggregated by population group, area or year. This has particular advantage when it comes to testing the effect of policies targeted at specific population groups, perhaps in specific geographical locations or on the basis of age or other demographics. It also allows assessment of variations in impact with respect to socio-demographic and geographical parameters as we have illustrated in this paper.

A second advantage, of particular importance for assessing health benefits of policy, is the potential to integrate evidence on a range of exposures and impacts including, for example, the outdoor and indoor environment as well as lifestyle behaviours, such as in travel behaviour and diet. Although our first implementation has so far been limited to outdoor air quality and deprivation, we are developing the model to cover other forms of exposure in order to better assess the impact of policies that affect a range of exposures, such as sectoral changes that arise in the transition to a low carbon economy. An important advantage of microsimulation is the possibility to incorporate agent based and behavioural modelling. This may, for example, be used to help model the distribution of the changes in patterns of behaviour and exposure in response to policies relating to transport infrastructure or pricing.

Another advantage of our particular model is that it provides a framework in which morbidity and mortality effects can be modelled in an integrated way. This is particularly important for some outcomes, including, as we illustrated, ischaemic heart disease morbidity, where impacts on disease survival may influence the size of the prevalent population with diagnosed disease, and thus have implications for healthcare provision and quality of life.

Microsimulation models, however, suffer from a common set of disadvantages (Schofield et al., 2018). They are data driven and so the predictions that they make are only as good as the data and the assumptions that feed into them. Difficulties may arise if trying to apply these types of methodologies to other countries or cities where health and population data are not so readily available. Developing countries may lack much of the necessary underlying data to support the microsimulation approach. This modelling approach is more computationally demanding than most, which not only slows the implementation of individual model runs but also limits the possibility of undertaking Monte Carlo analysis as a way of helping to characterize uncertainties in its outputs. Other methods can however be used to evaluate uncertainty in large scale models such as microsimulation. These include Global Sensitivity Analysis (Jaccard et al., 2018), Gaussian Process Emulation and Polynomial Chaos Expansion (Rajabi, 2019). Increases in HPC capacity in the future may allow sensitivity studies to be performed more easily (Jaccard et al., 2018). Moreover, because microsimulation relies on simulating stochastic processes, the outputs also reflect an element of random variation. These can be minimized by increasing the size of the simulated population, though with the penalty of increased computational time.

### 4.1 Comparison with other models

Table 2 summarizes other published microsimulation models of non-communicable diseases, which use a range of methods and risk factors. They include single state transition models that model mortality or morbidity independently. Our model is set up in a similar way to the IMPACT_NCD_ model (Kypridemos et al., 2016), although the IMPACT_NCD_ model was primarily investigating screening methods as opposed to environmental exposures. Other models have the ability to model co-morbidities that accumulate over time using aggregate health statistics (Kooiker and Boshuizen, 2018; Lymer et al., 2016; Walker et al., 2011).

**Table 2:** Microsimulation models.

Model	Country	Years	Heath outputs	Environmental risk factors	Reference
MicroEnv	London, England	2015–2065	Multiple. IHD morbidity/mortality	Air pollution	–
IMPACT NCD	England	to 2030	Cardiovascular disease and mortality	Screening methods	Kypridemos et al. (2016)
FORESIGHT	53 European countries	to 2030	Coronary heart disease, stroke, cancers	Obesity (Body Mass Index (BMI))	Webber et al. (2014)
UKHF-IC-PHE	England (local authorities)	2015–2035	Asthma, chronic obstructive pulmonary disease, coronary heart disease, stroke, type 2 diabetes, lung cancer	Air pollutants: PM_2.5_, NO_2_	Pimpin et al. (2018)
Basu	China & India	10 years	Disability adjusted life years	Blood pressure	Basu et al. (2016)
NCDMod	Australia	to 2025	Multiple chronic diseases	BMI, cholesterol, blood pressure and others	Lymer et al. (2016)
POHEM-CVD	Canada	2001-2021	Cardiovascular disease prevalence	BMI, cholesterol, blood pressure and others	Manuel et al. (2014)
DYNAMO-HIA	Netherlands	1989-2011	Lung and larynx cancer, stroke, diabetes, heart failure, coronary heart disease, COPD	Smoking	Kooiker and Boshuizen (2018)

MicroEnv, along with the joint UK Health Forum, Imperial College and Public Health England (UKHF-IC-PHE) model (Pimpin et al., 2018) are some of the first models to incorporate air pollution as a risk factor within a microsimulation model. There are several key differences between these models. The first, is that we use the GBD Results Tool to infer missing/unknown health transition probabilities, whilst the UKHF-IC-PHE model derives these using an in-house regression algorithm. The second, is that our model considers prevalence in an integrated manor within a multi-state model, as opposed to being modelled independently.

Given that air pollution varies widely between locations and also changes over time, a high spatial and temporal resolution is required to adequately assess health impacts locally. MicroEnv is able to output results at LSOA level which means that it has the potential to be further developed as a useful tool for local authorities as well as national government. The majority of other microsimulation models for non-communicable diseases report results at the national level (see Table 2), which restricts their application to national policy. The multitude of population, socio-economic and health data available for the UK, makes a high resolution analysis possible allowing the results to be mapped using GIS. Other countries, particularly in the developing world, do not have this luxury (Basu et al., 2016) and assumptions need to be made where detailed data is not available. The HPC used to run MicroEnv allows the handling of large amounts of data, as well as simulation over a long time frame with annual health calculations.

A challenge when projecting the future burden of disease is to account for the ways in which the calculations, risk factors (e.g. air pollution) and subjects (people) change with time. In the current version of MicroEnv, the calculations (i.e. the health transition matrix for a person of a particular age) are static over time. It is possible to change the all-cause mortality probability to the ONS projection (up until 2062), as is done in IMPACT_NCD_ (Kypridemos et al., 2016), however, projections for case fatality and incidence are not available and would need to be estimated. Projections are also likely to have larger uncertainties for predictions made further into the future. We must also consider how the confounding risk factors evolve over time. Birth and migration are factors that microsimulation models are able to take into account. Whilst the majority of the models in Table 2 include births, only the POHEM-CVD (Manuel et al., 2014) includes immigration and emigration. These factors may significantly affect the results of models, particularly at local levels. London, for example, has high levels of immigration of people aged 20–30, whilst those above the age of 30 tend to move out of the city or to another city altogether (ONS, 2014).

## 5 Conclusions

The SDGs call for urgent action to combat climate change, reduce population exposure to environmental hazards such as air pollution, and to improve population health in urban environments. There are opportunities to select policies and developmental paths that will provide both environmental improvement and optimise health co-benefits. Modelling tools such as microsimulation have an important role in determining the most cost-effective and impactful methods of achieving such environmental and public health targets, enabling a range of scenarios to be evaluated for both health and sustainability outcomes. As such, modelling is capable of helping to support the achievement of SDGs, as well as evaluating other environmental strategies that help to facilitate the transition to a low carbon economy.

We have described a microsimulation model that simulates the effects of environmental exposures on mortality and morbidity in an integrated manor. The framework is capable of quantifying the health impact of multiple environmental risks at high spatial resolution. It is therefore well suited to assessing health impacts, and how these are distributed across different population demographics such as by gender or deprivation. Its disadvantage is that it is computationally demanding and therefore not readily adapted to being a rapid response decision-support tool. However, the outputs generated by the model can be used to inform policy development and in supporting healthy and sustainable urban development in line with the ambitions of the SDGs.

## Declaration of competing interest

The authors declare that they have no known competing financial interests or personal relationships that could have appeared to influence the work reported in this paper.

## Supplementary material

Appendices A-D provide additional information to support the paper as referenced to from within the text; Appendix A: Bayes theory, Appendix B: Health data, Appendix C: Model input data, Appendix D: Results for alternative scenarios. Image 1
