# Supplementary material for: MicroEnv: A microsimulation model for quantifying the impacts of environmental policies on population health and health inequalities
Source: Sci Total Environ. 2019 Dec 20;697:134105. doi: 10.1016/j.scitotenv.2019.134105 (PMC7212697; doi:10.1016/j.scitotenv.2019.134105)
Supplement: Supplementary file 1 — Appendices A-D provide additional information to support the paper as referenced to from within the text; Appendix A: Bayes theory, Appendix B: Health data, Appendix C: Model input data, Appendix D: Results for alternative scenarios. [file mmc1.docx]

**Appendix A: Bayes theory**

The GBD Results Tool data is published as a rate per population. Our three-state model requires these, per population, probabilities to be converted to conditional probabilities to reflect individuals being in either a healthy or diseased state. Here we present the full workings for the derivation for the incidence probability from a healthy state $P\left( I | H1 \right)$.

Bayesian probability theory states that for any events X and Y:

$$P\left( X \cap Y \right)=P\left( X \right)P\left( Y | X \right)=P\left( Y \right)P(X|Y)$$

Where:

- $P$ is probability
- $P(X\cap Y)$ probability of events X and Y both happening
- $P(X|Y)$ probability of event X happening conditional on event Y

Now

$$P\left( X \cap Y \right)=P\left( X \right)P\left( Y | X \right)=P\left( Y \right)P(X|Y)$$

means that

$$P\left( X \right)P\left( Y | X \right)=P\left( Y \right)P(X|Y)$$

and hence Bayes rule

$$P\left( Y | X \right)=\frac{P\left( X | Y \right)P(Y)}{P(X)}$$

In the above equation let:

$$Y=I$$

$$X=H1$$

where $I$ is IHD incidence and $H1$ is non-IHD prevalence (i.e. probability of being healthy).

Then

$$P\left( I | H1 \right)=\frac{P\left( H1 | I \right)P(I)}{P(H1)}$$

but probability of non-IHD prevalence = 1 – probability of IHD:

$$P\left( H0 \right)=1-P(H1)$$

We can prove that $P\left( H1 | I \right)=1$, since individuals are only able to become incident if in a healthy state (H1). Using the normalising condition: $P\left( H1 | I \right)+P\left( H2 | I \right)=1$. Where $P\left( H2 | I \right)=0$; since disease incidence and being already in a diseased state, H2, are mutually exclusive.

This allows the incidence probability to be simplified giving:

$$P\left( I | H1 \right)= \frac{P(I)}{(1-P\left( H2 \right))}$$

**Appendix B: Health data**

This appendix provides further details of the health data used as input to the model. For Ischaemic Heart Disease (IHD) prevalence and mortality, a comparison between data from local health surveys (e.g. the ONS and Health Survey for England) and outputs from the Global Burden of Disease (GBD) Results Tool is performed.

**B1. All-cause mortality rates**

All-cause mortality, data from the Office for National Statistics (ONS, 2017). The ONS provides both period and cohort projections by single year of age and sex for the UK. Although the model is able to take these projections into account, we opted to use fixed (2016) values for mortality rates within MicroEnv. Figure B1 shows all-cause mortality rates used in the model by age and sex.


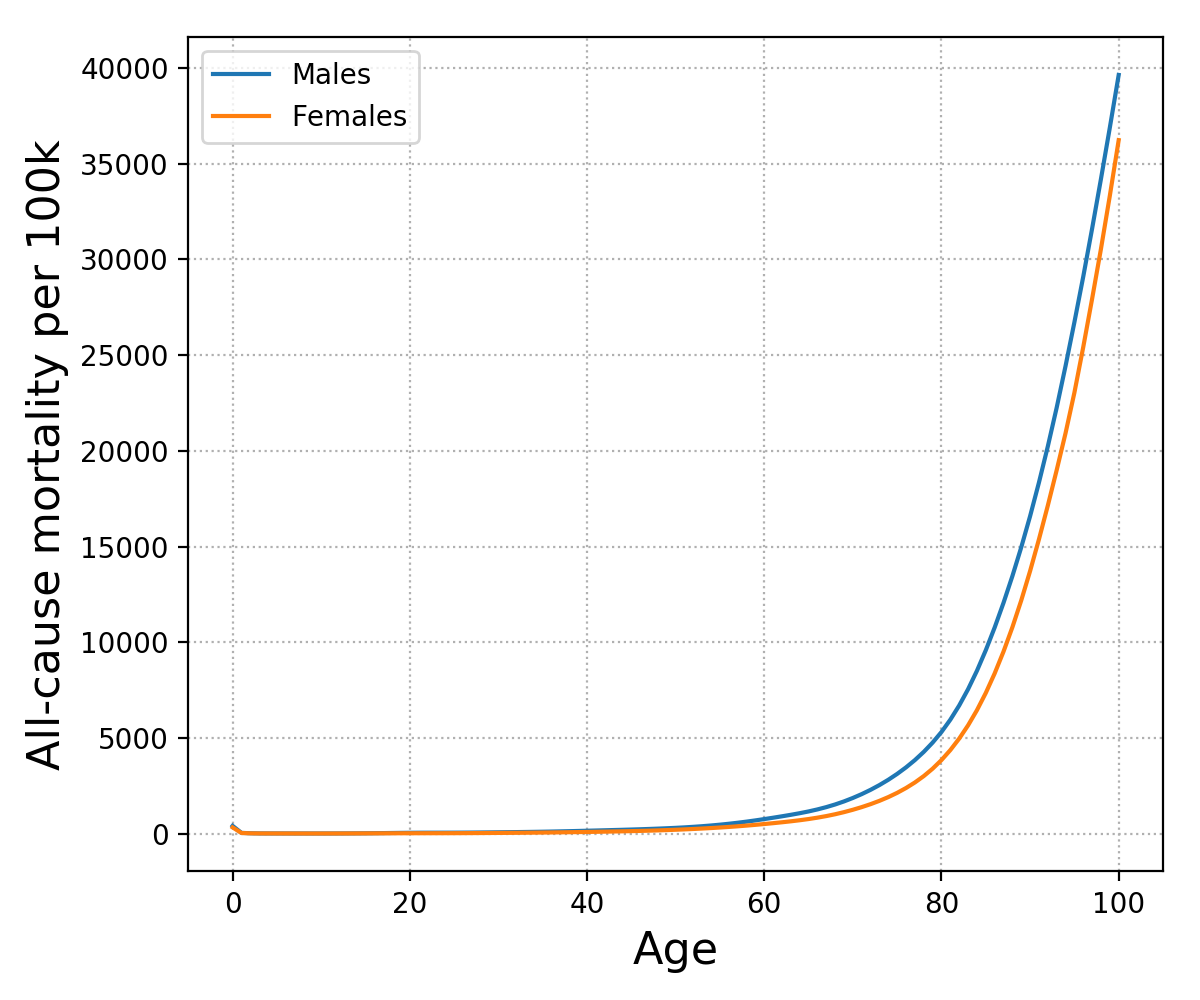


Figure B1. UK all-cause mortality rates (per 100k) used in MicroEnv by single year of age and sex.

**B2. Ischaemic Heart Disease incidence, prevalence and mortality rates**

IHD incidence, prevalence and mortality data used within MicroEnv is obtained from the GBD Results Tool (Institute for Health Metrics and Evaluation, 2019). Data by 5-year age band and sex for the UK (2016) was used within our model. In order to compare the GBD outputs to the latest health survey data, the graphs in this appendix show data for England (2017). Figures B2, B3 and B4 show incidence, prevalence and mortality data, respectively. For the incidence data, it is not possible to make a comparison with health survey data, since the Health Survey for England (HSE) (NHS Digital, 2019) report incidence in terms of hospital inpatient admissions rather than new cases of IHD. The comparison between the GBD Results Tool prevalence outputs and HSE data shows that there is a reasonable agreement, except at older ages. Since the results we show in the paper are for people of working age (15-64), this should not have much influence on our results. A similar discrepancy is observed for IHD mortality when comparing to ONS data downloaded from the nomis online tool (ONS, 2019).


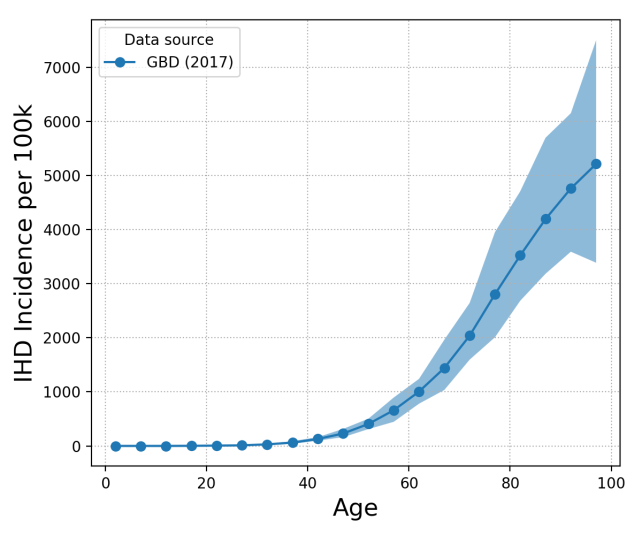

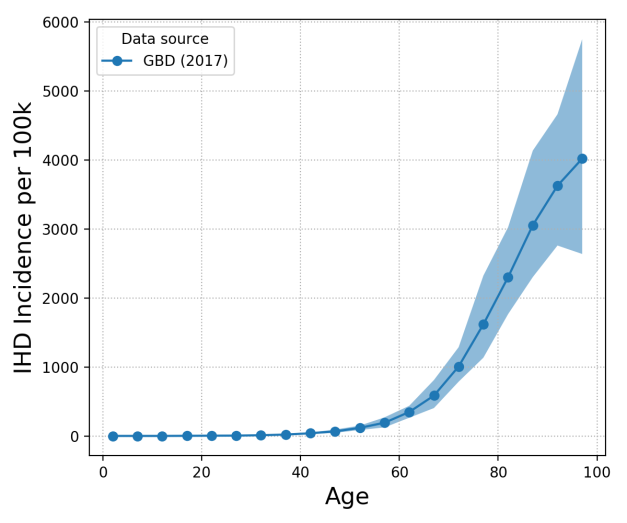


Figure B2. IHD incidence rates (per 100k) in England (2017) by age as output by the GBD Results Tool for males (left) and females (right). Error bands represent 95% confidence interval reported by GBD Results Tool.


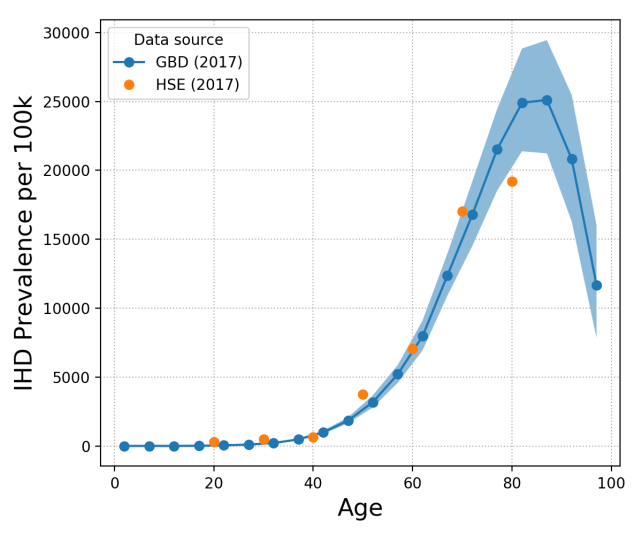

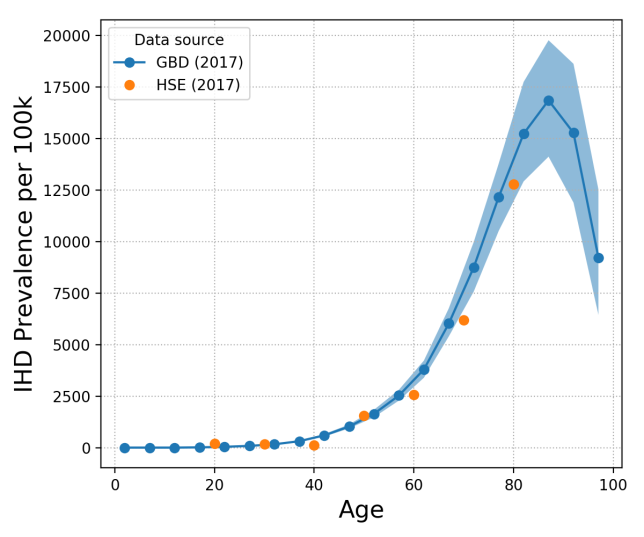


Figure B3. IHD prevalence rates (per 100k) in England (2017) by age as output by the GBD Results Tool compared to HSE data for males (left) and females (right). Error bands represent 95% confidence interval reported by GBD Results Tool.


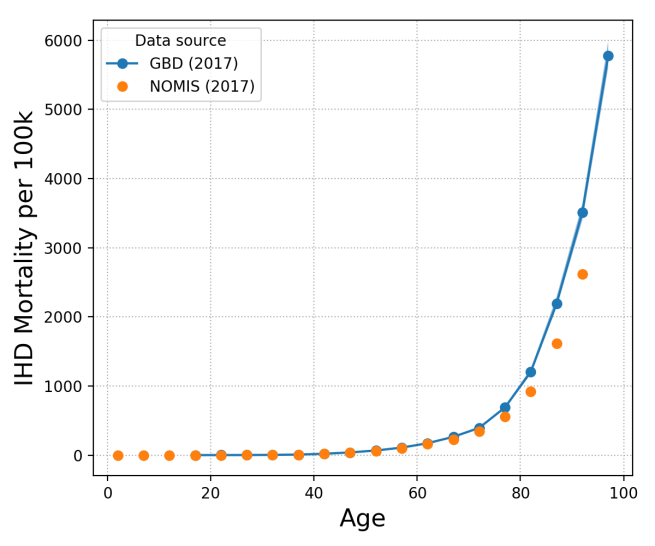

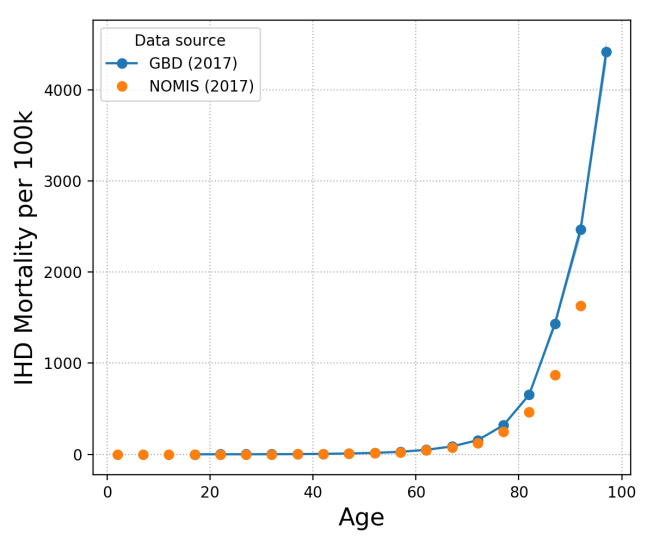


Figure B4. IHD mortality rates (per 100k) in England (2017) by age as output by the GBD Results Tool compared to nomis data from the ONS for males (left) and females (right).

**Appendix C: Model input data**

This appendix provides details of the inputs that have been used within the model and includes information on the risk factors (PM2.5 and deprivation). The population data used to initialise the model and the fertility data used are also described.

**C.1 Population data**

The population data used within MicroEnv to generate the *true* population of Greater London is from the Office for National Statistics (ONS, 2018a). Data is available for the 2015 population by single year of age and by sex for each LSOA in Greater London. Figure C1 maps the population of each LSOA modelled.


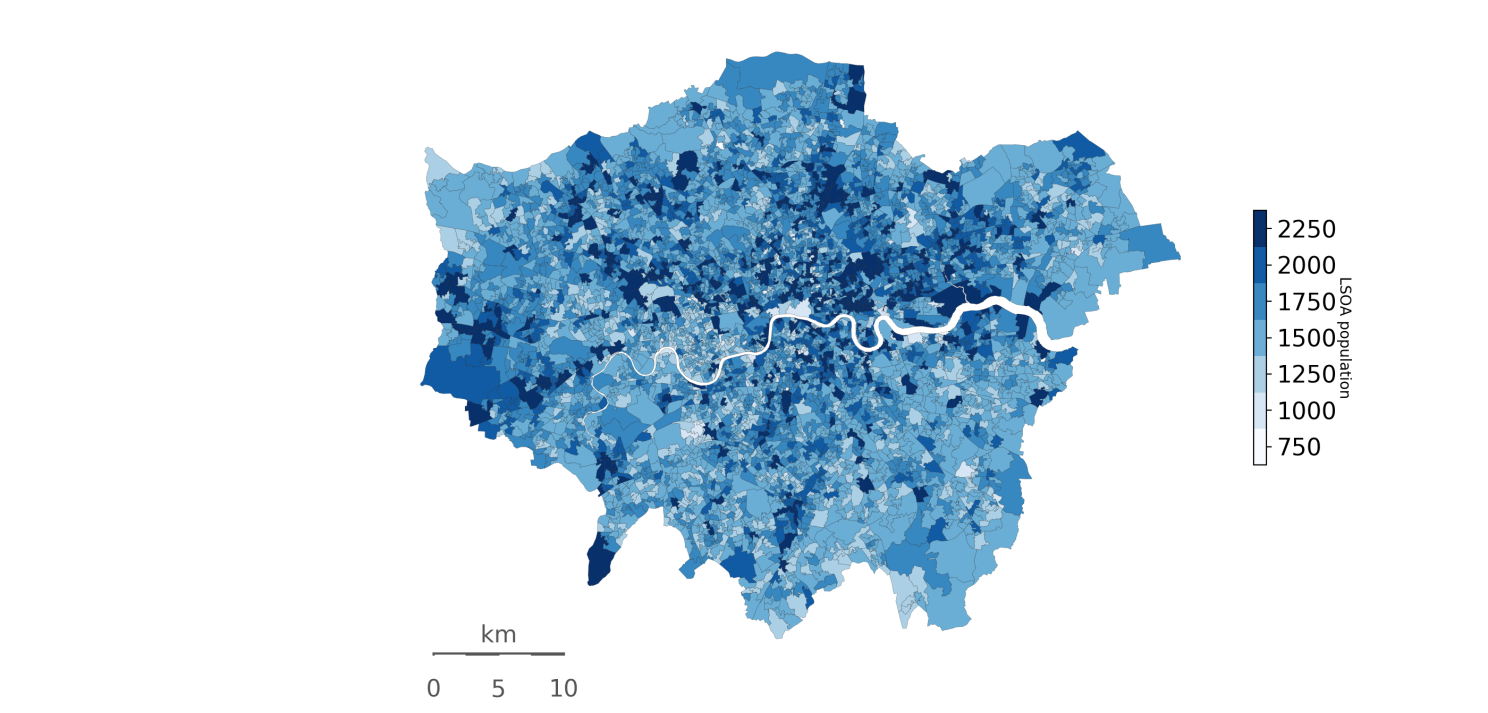


Figure C1. Map of population (2015) by LSOA for Greater London.

**C2. Socio-economic deprivation**

The socio-economic deprivation of individuals within each LSOA is defined using the 2015 decile of the Index of Multiple Deprivation (IMD). These are reported by the UK Department for Communities and Local Government (DCLG) (Department for Communities and Local Government, 2015). Figure C2 provides a map of IMD by LSOA for Greater London.


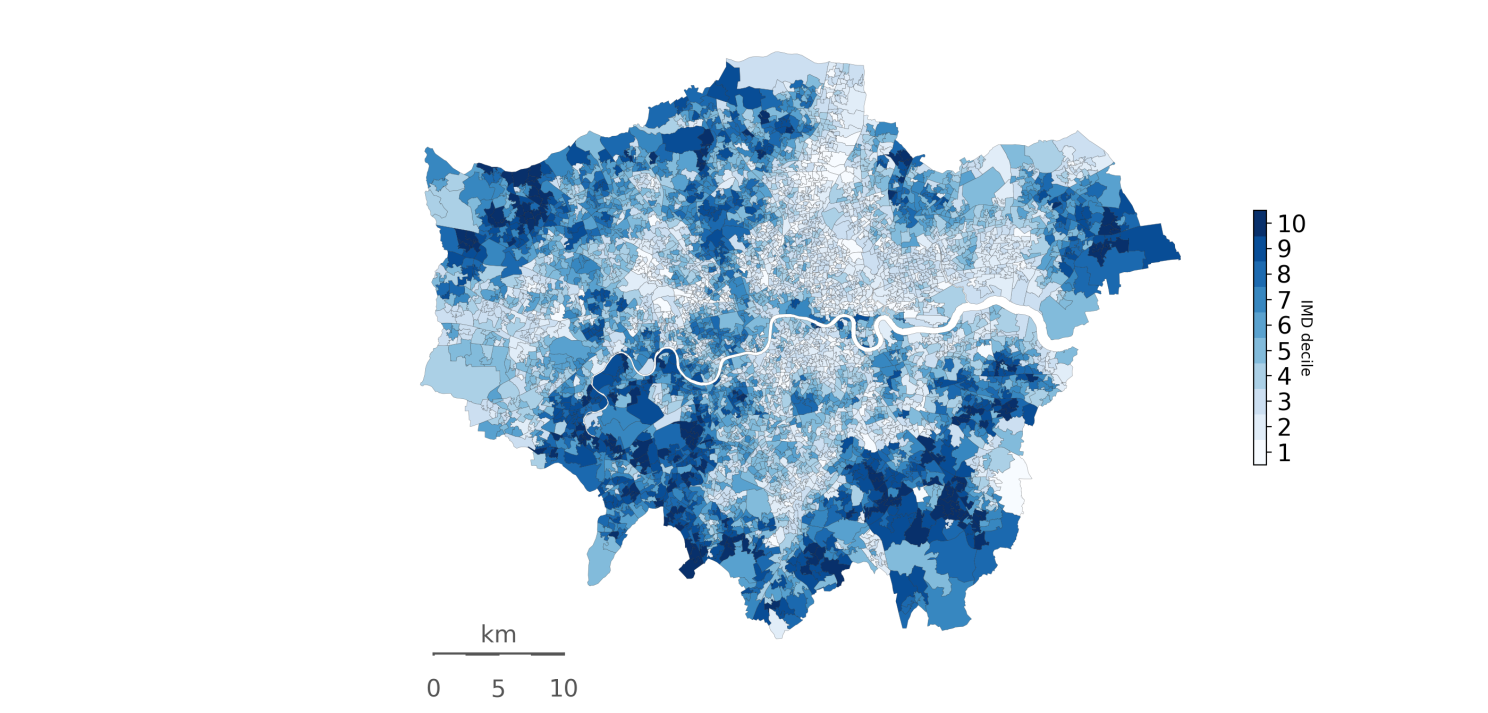


Figure C2. Map of IMD (2015) by LSOA for Greater London. A lower deprivation decile represents a more deprived area.

**C3. Air pollution (PM_2.5_) modelled data**

Modelled PM_2.5_ data for 2014 has been used within MicroEnv (Ricardo Energy & Environment, 2017). This was based on air pollution modelled at 1 x 1 km grid square for the UK using the air dispersion model ADMS and emissions estimates from the UK National Atmospheric Emissions Inventory 2014 (NAEI, 2014). The modelled air pollution was linked to provide an average for each LSOA using GIS methods. Figure C3 maps the modelled annual average PM_2.5_ concentration in Greater London.


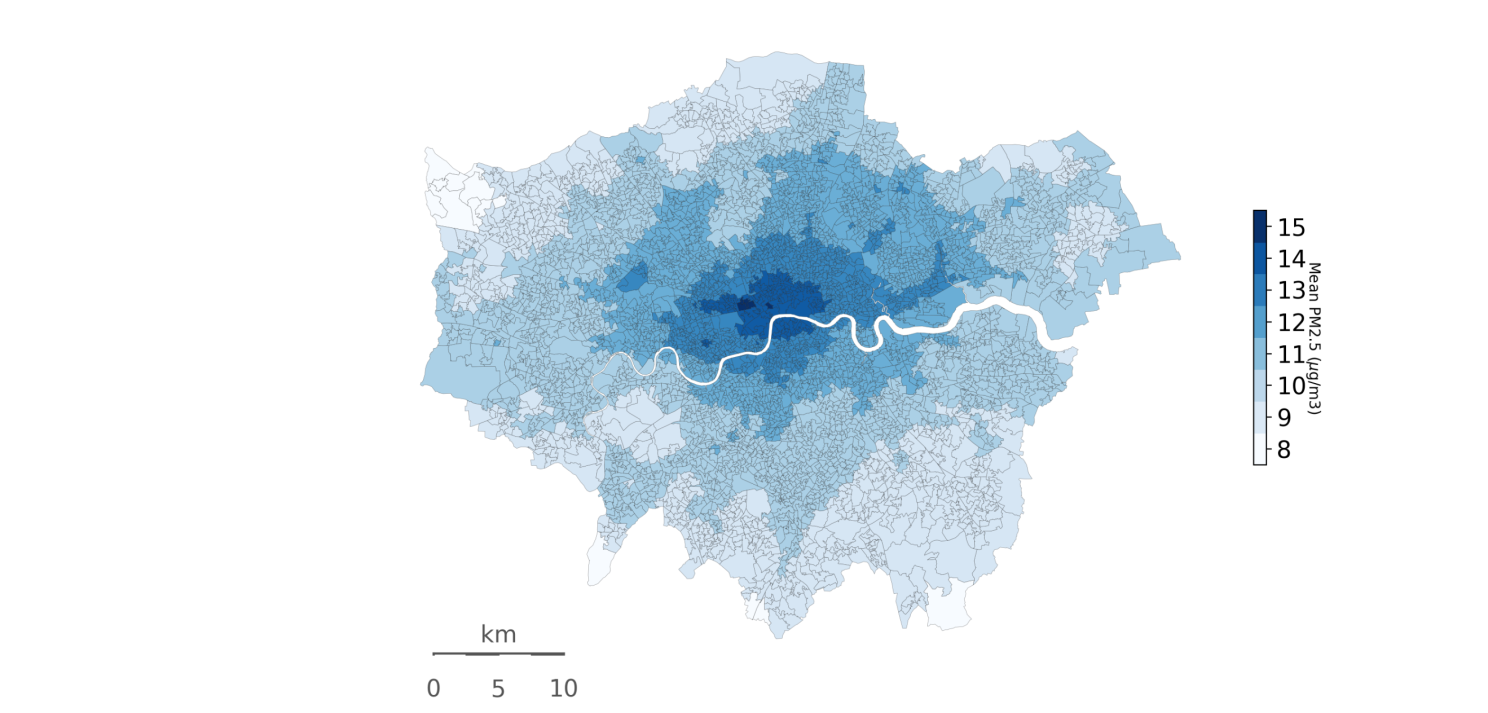


Figure C3. Map of modelled PM_2.5_ in 2014 by LSOA for Greater London.

**C4. General Fertility Rates (GFRs)**

The number of new births simulated in the model each year is calculated using local authority level General Fertility Rate date (the number of live births per 1,000 females aged 15-44 per year) (ONS, 2018b). At the end of each year, for each LSOA, the number of females aged 15-44 is multiplied by the GFR to estimate the number of new births. Newborns are assigned gender with a 50/50 probability. Figure C4 maps the GFRs by local authority as used within the model.


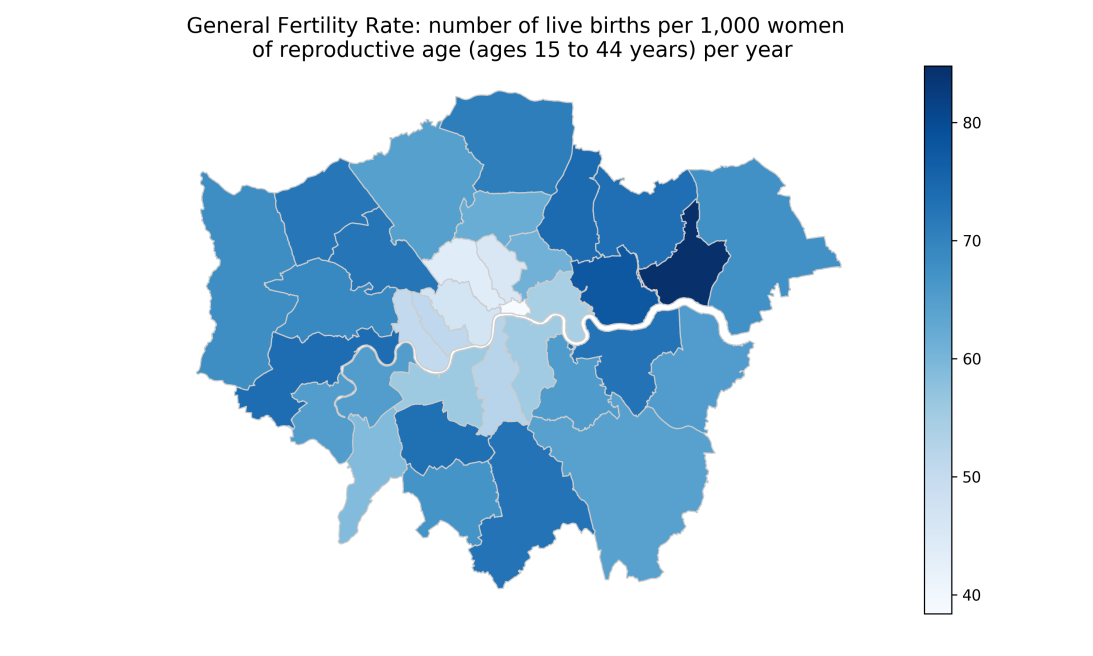


Figure C4. General Fertility Rate mapped at local authority level for Greater London.

**Appendix D: Results for alternative scenarios**

Here we provide illustrative maps of the impact of two air pollution reduction scenarios on IHD prevalence and all-cause mortality with respect to the base case (2014) scenario. These two scenarios are as follows:

1. WHO: Compliance with World Health Organisation guidelines (WHO, 2005) (i.e. ambient outdoor PM_2.5_ annual mean does not exceed 10 µg/m^3^ – only effects LSOAs where PM_2.5_ > 10 µg/m^3^)
2. NECD (National Emissions Ceiling Directive): UK emission reductions in line with EU Directive 2016/2284/EU resulting in the baseline concentration reducing by 3.6 µg/m^3^ across Greater London (GLA, 2017)

**
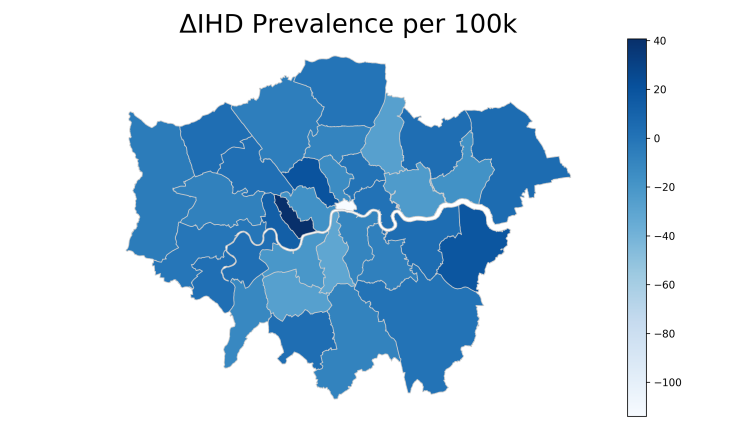

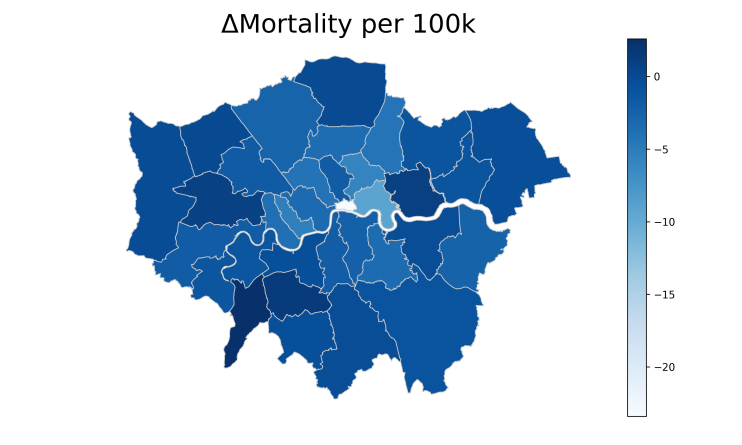
**

Figure D1. Illustration of Local Authority-level outputs for Greater London: change in IHD prevalence (left) and all-cause mortality (right) rates per 100k working age (15-64) population resulting from the WHO scenario (A) with respect to the base case scenario (PM_2.5_ concentrations at 2014 level). The results shown are averaged over the 2018-2050 modelling period.

**
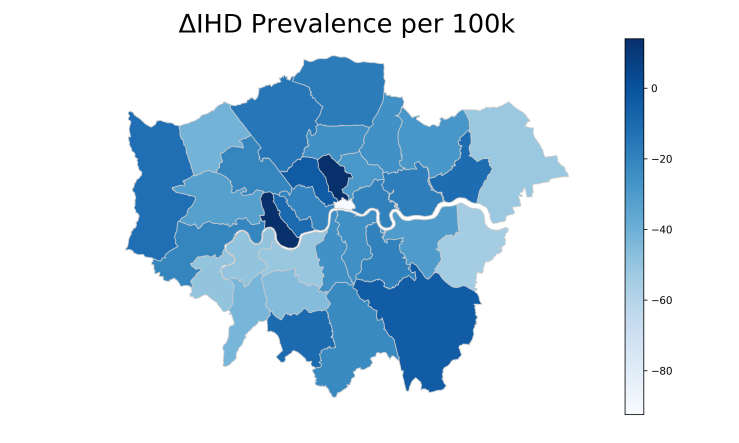

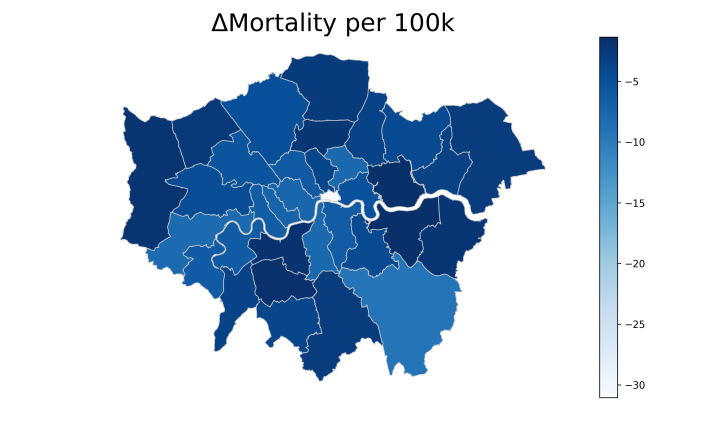
**

Figure D2. Illustration of Local Authority-level outputs for Greater London: change in IHD prevalence (left) and all-cause mortality (right) rates per 100k working age (15-64) population resulting from the NECD scenario (B) with respect to the base case scenario (PM_2.5_ concentrations at 2014 level). The results shown are averaged over the 2018-2050 modelling period.

**References**

Department for Communities and Local Government, 2015. English Indices of Deprivation 2015. URL https://www.gov.uk/government/statistics/english-indices-of-deprivation-2015 (accessed 07.18.19).

NHS Digital, 2019. Health Survey for England - Health, social care and lifestyles. URL https://digital.nhs.uk/data-and-information/publications/statistical/health-survey-for-england/2017 (accessed 07.18.19).

Institute for Health Metrics and Evaluation, 2019. GHDx: GBD Results Tool [WWW Document]. URL http://ghdx.healthdata.org/gbd-results-tool (accessed 3.7.19).

NAEI, 2014. UK NAEI - National Atmospheric Emissions Inventory. URL http://naei.beis.gov.uk/ (accessed 07.18.19).

ONS, 2018a. Lower layer Super Output Area population estimates [WWW Document]. URL https://www.ons.gov.uk/peoplepopulationandcommunity/populationandmigration/populationestimates/datasets/lowersuperoutputareamidyearpopulationestimates (accessed 3.7.19).

ONS, 2018b. Births in England and Wales [WWW Document]. Office for National Statistics. URL https://www.ons.gov.uk/peoplepopulationandcommunity/birthsdeathsandmarriages/livebirths/bulletins/birthsummarytablesenglandandwales/2017 (accessed 3.7.19).

ONS, 2017. Past and projected data from the period and cohort life tables, 2016-based [WWW Document]. URL https://www.ons.gov.uk/peoplepopulationandcommunity/birthsdeathsandmarriages/lifeexpectancies/bulletins/pastandprojecteddatafromtheperiodandcohortlifetables/2016baseduk1981to2066 (accessed 3.7.19).

ONS, 2017. Nomis. URL https://www.nomisweb.co.uk/default.asp (accessed 07.18.19)

Ricardo Energy & Envinoment, 2017. Technical report on UK supplementary assessment under The Air Quality Directive (2008/50/EC), The Air Quality Framework Directive (96/62/EC) and Fourth Daughter Directive (2004/107/EC) for 2015
